# Morpho-Functional Analyses Demonstrate That Tyrosol Rescues Dexamethasone-Induced Muscle Atrophy

**DOI:** 10.3390/jfmk9030124

**Published:** 2024-07-17

**Authors:** Sara Salucci, Sabrina Burattini, Ilaria Versari, Alberto Bavelloni, Francesco Bavelloni, Davide Curzi, Michela Battistelli, Pietro Gobbi, Irene Faenza

**Affiliations:** 1 Department of Biomedical and NeuroMotor Sciences, University of Bologna, 40126 Bologna, Italy; ilaria.versari4@unibo.it (I.V.); francesco.bavelloni25@gmail.com (F.B.); irene.faenza2@unibo.it (I.F.); 2 Department of Biomolecular Sciences, University of Urbino Carlo Bo, 61029 Urbino, Italy; sabrina.burattini@uniurb.it (S.B.); michela.battistelli@uniurb.it (M.B.); pietro.gobbi@uniurb.it (P.G.); 3 Laboratory of Experimental Oncology, Istituto di Ricovero e Cura a Carattere Scientifico (IRCCS), Istituto Ortopedico Rizzoli, 40136 Bologna, Italy; alberto.bavelloni@ior.it; 4 Department of Humanities, Movement, and Education Sciences, University “Niccolò Cusano”, 00166 Rome, Italy; davide.curzi@unicusano.it

**Keywords:** olive oil flavonoid, muscle atrophy, mitochondria and autophagy involvement, lysosomal functionality, confocal and electron microscopy

## Abstract

Prolonged exposure to high dosages of dexamethasone, which is a synthetic glucocorticoid and a well-known anti-inflammatory drug, may lead to an increase in reactive oxygen species production, contributing to muscle wasting. The prevention of muscle atrophy by ingestion of functional foods is an attractive issue. In the last decade, natural antioxidant compounds have been increasingly investigated as promising molecules able to counteract oxidative-stress-induced muscle atrophy. Recently, we have demonstrated the antioxidant properties of two main olive oil polyphenols also known for their anticancer and anti-inflammatory activities in different cell models. Here, the preventive effect of tyrosol on dexamethasone-induced muscle atrophy has been investigated by means of morpho-functional approaches in C2C12 myotubes. Dexamethasone-treated cells showed a reduced fiber size when compared to control ones. While long and confluent myotubes could be observed in control samples, those exposed to dexamethasone appeared as immature syncytia. Dysfunctional mitochondria and the accumulation of autophagic vacuoles contributed to myotube degeneration and death. Tyrosol administration before glucocorticoid treatment prevented muscle wasting and rescued mitochondrial and lysosomal functionality. These findings demonstrate that tyrosol attenuates dexamethasone-induced myotube damage, and encourage the use of this natural molecule in preclinical and clinical studies and in synergy with other functional foods or physical activity with the aim to prevent muscle atrophy.

## 1. Introduction

Muscle wasting is a condition that occurs during aging or can be a physiological response to fasting or malnutrition. Furthermore, it plays a crucial role in many pathologies, including neuromuscular diseases, chronic obstructive pulmonary disorder, cancer-associated cachexia, diabetes, renal failure, cardiac failure, Cushing syndrome, sepsis, burns, and trauma [1,2]. Skeletal muscle is a plastic tissue able to adapt its size and function to external and internal stimuli, and its homeostasis is strictly linked to the balance between protein synthesis and degradation, which depends on muscle fiber size and contractile function [3]. It is known that rapid loss of muscle mass is due to excessive protein breakdown, which is often accompanied by reduced protein synthesis.

Glucocorticoids, which have anti-inflammatory and immune-suppressing properties, are commonly used in the treatment of several diseases, such as rheumatoid arthritis, systemic lupus erythematosus, bronchial asthma, adult dyspnea syndromes, autoimmune diseases, and others [4,5,6]. However, high doses or prolonged use of these drugs can cause many side effects including muscle weakness and atrophy [7,8]. Chronic or excessive exposure to glucocorticoids induces muscle atrophy through ubiquitin-proteasome and lysosomal pathways.

In skeletal muscle, glucocorticoids up-regulate the expression of myostatin and muscle-specific E3 ubiquitin ligases such as F-box only protein 32 (atrogin-1) and muscle RING-finger protein-1 (MuRF-1) [9]. Furthermore, when muscle cells are exposed to dexamethasone (DEXA), which is a synthetic glucocorticoid widely used as an atrophy inducer in both in vivo and in vitro models [10], reactive oxygen species (ROS) production increases, resulting in mitochondrial dysfunctions, which, as known, contribute to muscle mass loss [11].

Significant advances have been made to highlight the mechanisms involved in regulating muscle loss in diseases. However, effective pharmacological treatments for preventing or delaying muscle atrophy are currently unavailable [12]. Recently, various nutraceutical strategies have been described to counteract muscle alterations induced by oxidative stress, and, consequently, to preserve muscle homeostasis. Among these nutrients, olive oil, an integral ingredient of the ‘Mediterranean diet’, has shown several beneficial effects against cancer, metabolic syndrome, heart defects, and muscle dysfunctions too. In this regard, epidemiological studies on the Mediterranean population have demonstrated that a regular intake of olive oil has several beneficial health effects [13,14]. In humans adhering to the Mediterranean diet, a reduction in the incidence of chronic degenerative diseases, major cardiovascular events, type 2 diabetes mellitus, and some types of cancer has been evidenced together with an improvement in cognitive function [13]. Olive oil’s favorable health effects are due to a valuable source of highly abundant unsaturated fatty acids and minor compounds like fat-soluble vitamins, chlorophylls, phytosterols, and polyphenols [15]. According to numerous investigations, these effects are mostly attributable to the main secorroid derivatives, such as oleuropein, oleocanthal, and oleacein, and the simple phenols hydroxytyrosol (Hyt) and tyrosol (Tyr). These latter phenolic compounds are known for their antioxidant and anti-inflammatory properties [15], and the activity of Hyt in preventing muscle loss has already been described [16,17,18]. For instance, Hyt in association with other nutrients can ameliorate disuse-induced muscle atrophy, and it can counteract mitochondrial dysfunctions induced by oxidative stress in skeletal muscle cells [16,17,18]. Since then, no studies have reported the effects of Tyr on muscle disorders and wasting. Nevertheless, Tyr can be considered an interesting compound even if it could have a lower antioxidant capacity than Hyt [15] due to the absence of an ortho (italic)-diphenol group in its chemical structure. However, over time, it can maintain its antioxidant activity if compared to other natural flavonoids that are characterized by a drastic reduction of their antioxidant effect, and sometimes even become pro-oxidants [15]. Recently, some researchers demonstrated Tyr action in inhibiting oxidative damage and preventing cell death of L6 muscle cells through the regulation of extracellular signals such as c-Jun N-terminal kinase and p38 MAPK [19]. Therefore, understanding the role of this antioxidant in maintaining muscle mass could open interesting scenarios in the field of muscle atrophy and degeneration. For that reason, in this study, we determined by means of morpho-functional analyses whether Tyr could mitigate muscle loss in murine myotubes exposed to DEXA, which is a known glucocorticoid drug, used in vitro to induce an atrophic phenotype [20]. We investigated the effects of Tyr alone and its administration before DEXA treatment on skeletal muscle morphology, with particular attention on mitochondria, autophagy, and lysosomal involvement.

## 2. Materials and Methods

### 2.1. Cell Culture

C2C12 (it is a commercial cell model by MilliporeSigma, Waltham, MA, USA) murine undifferentiated cells were grown in flasks or on coverslips in Petri dishes using Dulbecco’s Modified Eagle’s Medium (DMEM; Gibco, Thermo Fisher Scientific, Waltham, MA, USA) supplemented with 10% heat-inactivated fetal bovine serum (FBS; Sigma-Aldrich, F2442, Waltham, MA, USA), 2 mM glutamine (Sigma-Aldrich, G8540), and 1% antibiotics (Sigma-Aldrich, P4333) [21]. Upon reaching approximately 80% confluence, myotube formation was induced by replacing growth medium with differentiation medium supplemented with 1% FBS [22]. Cells were maintained at 37 °C in a humidified atmosphere with 5% CO_2_, and monitored daily with a Nikon Eclipse TE 2000-S inverted Microscope equipped with a digital DN 100 Nikon system.

**Scheme 1:**
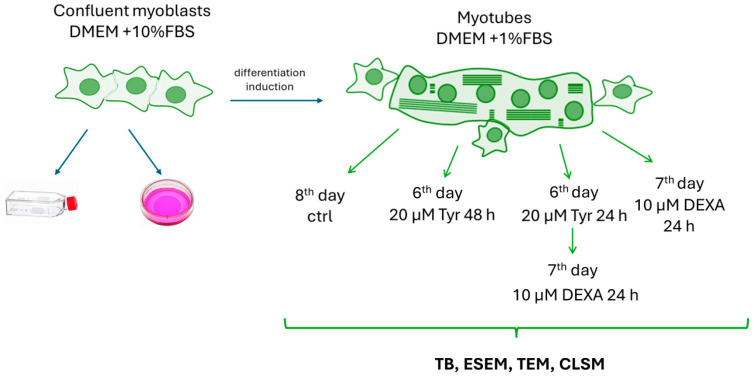
Experimental design.

Treatments were performed after six days of differentiation by treating cells with Tyr before DEXA (Sigma-Aldrich, D2915) administration or after seven days of differentiation by exposing myotubes to DEXA alone, as detailed in Scheme 1.

### 2.2. Treatments and Trypan Blue (TB) Exclusion Assay

Various Tyr concentrations (5 µM, 10 µM, 20 µM, 40 µM, and 80 µM) were tested against 10µM DEXA-induced damage. This latter dosage is the one indicated for inducing in vitro muscle atrophy [20]. A TB (Thermo Fischer Scientific, 15250061) cell viability exclusion assay [23] allowed investigation of the DEXA effect on myotube viability and understanding of the correct Tyr concentration able to prevent DEXA-induced cell damage. In detail, cells were washed and suspended (1.0 × 10^5^ cells/mL) in a solution of 1× PBS (Phosphate Buffer Saline; Gibco™ by Life Technologies, Waltham, MA, USA). Fifty (50) microliters of the cell suspension were mixed with an equal volume of 0.4% TB. Ten (10) microliters of the solution were transferred to a hemocytometer, which automatically counted both viable (clear) and dead (blue) cells. The percentage cell viability was obtained by counting the number of live cells and dividing it by the total number of counted cells (clear and blue). The best Tyr dose against DEXA was 20 µM. Thus, myotubes at the end of the sixth day of differentiation were pre-treated with 20 µM Tyr and then exposed to DEXA for a further 24 h. At the end of the seventh day of differentiation, some cells were exposed to DEXA alone. Furthermore, the 20 µM polyphenol dose administrated at the end of the sixth day of differentiation was evaluated by TB after 48 h of treatment to investigate its potential cytotoxic effect in our murine culture model. Then, control and treated myotubes were processed for morpho-functional analyses.

### 2.3. Environmental Scanning Electron Microscopy (ESEM)

Myotubes grown on coverslips were dried and mounted on conventional SEM stubs (Agar Scientific, Stansted, Essex CM24 8GF United Kingdom), and then observed with a FEI QUANTA 200 ESEM (mode: low vacuum; detector: secondary electrons; high voltage: 30 kV).

For all experimental conditions, the transversal diameter of one hundred myotubes was calculated. Diameters from all experimental conditions were compared with each other to understand the effect of DEXA on myotube size and to evaluate the potential protection of Tyr in the preservation of muscle mass [24].

### 2.4. Transmission Electron Microscopy (TEM)

Differentiated cells were grown on coverslips in Petri dishes to obtain monolayers. Then, myotubes were rinsed with PBS and immediately fixed “in situ” with 2.5% glutaraldehyde (Thermo Fisher Scientific, 119980250) in 0.1 M phosphate buffer for 45 min. Then, cells were post-fixed in 1% OsO_4_ (Agar Scientific) for 1 h and alcohol-dehydrated as described by Codenotti and coworkers [25]. Monolayers were covered with capsules full of araldite resin (Durcupan, Sigma-Aldrich); coverslips were then crushed and removed in liquid nitrogen.

Thin sections (~0.3 µm), after UranyLess (Electron Microscopy Sciences, Hatfield, PA, USA) and lead citrate (Electron Microscopy Sciences) staining, were observed with an electron microscope [26].

### 2.5. Confocal Laser Scanning Microscopy (CLSM)

For CLSM, cells were grown on a coverslip in a six-well Petri dish, and the following fluorescent reactions were carried out:

- LC3

A Premo™ Autophagy Sensor LC3B-GFP (Life Technologies, Carlsbad, CA, USA) was used to detect LC3 protein localization; cells were transduced following the protocol provided by the datasheet [3]. This fluorochrome allows the visualization of autophagosomes and autophagolysosomes, which can be detected through fluorescent green puncta.

- AO

AO (Invitrogen, CA1301, Waltham, MA, USA) is a pH-sensitive dye used to detect acidic vesicular organelle formation, and it is considered a well-proven staining technique to monitor lysosomal membrane stability, mainly through fluorescence microscopy [3]. AO at the highest concentrations stains DNA, while at the lowest concentrations, in an acid environment, AO emits red fluorescence with an intensity proportional to the degree of acidity and/or to the acidic compartment volume. When the lysosomal proton gradient is lost, leakage of AO into the cytosol can be detected as a concomitant increase in green cytosolic staining and a loss of red lysosomal staining.

Cells were washed and re-suspended in 0.5 mL of medium and then stained with 75 ng/mL of AO for 15 min at 37 °C.

- JC1

JC-1 (5,5′,6,6′-tetrachloro1,1′,3,3′-tetramethylbenzimidazolylcarbocyanine iodide; Invitrogen, T3168) was used to monitor mitochondrial membrane potential. The ratio of green/red fluorescence is independent from mitochondrial density, size, and shape, and it only correlates to the mitochondrial membrane potential. Therefore, JC-1 dye monomers form red fluorescent “J-aggregates” in the presence of a high mitochondrial potential, while a low potential corresponds to green fluorescent “J-aggregates”.

C2C12 differentiated cells from different treatment groups were washed in PBS and incubated with JC-1 (1 μM) in DMEM at 37 °C for 20 min.

All samples were observed with a Leica TCS-SP5 CLSM [24] connected to a DMI 6000 CS Inverted Microscope (Leica Microsystems CMS GmbH); LC3 (Exc/Em = 488/522); AO (Exc 488 nm, emission 480–560 nm (monomers), and 650 nm (stacks)); JC-1 (Exc 488 nm, emission 530 nm for dye monomers, and 590 nm for J-aggregates). Densitometric analysis of LC3 or JC1 immunofluorescence was performed with ImageJ 1.54j Software (National Institutes of Health).

### 2.6. Statistical Analyses

Data obtained from TB, ESEM, and CLSM are presented as mean ± standard deviation (SD) and derived from at least three independent experiments.

Data were analyzed using one-way ANOVA, and post-hoc analyses were conducted using Tukey’s multiple comparisons test to identify specific differences between treatment groups.

Results were considered statistically significant when *p* < 0.05 (*) and highly significant when *p* < 0.005 (**).

## 3. Results

### 3.1. TB Assay

**Figure 1:**
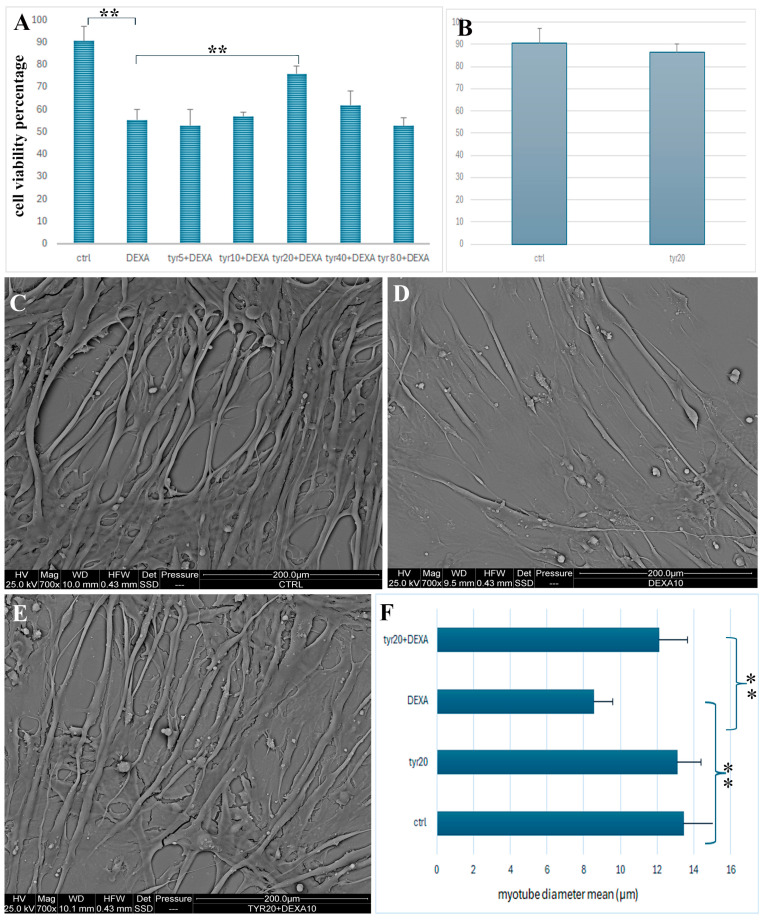
Cell viability percentage evaluated through TB assay in control cells, in cells exposed to DEXA, and cells pre-treated with various Tyr (5 µM, 10 µM, 20 µM, 40 µM, and 80 µM) dosages before DEXA administration (**A**). Graph shows TB assay of control cells and those treated with Tyr 20 μM for 48h (**B**). ESEM images show control myotubes (**C**) and those exposed to DEXA (**D**) or pretreated with Tyr and then exposed to DEXA (**E**). In (**F**) graph shows the mean of myotube diameter in all experimental conditions. *p*-value < 0.005 (**, highly significant): ctrl vs. DEXA and DEXA vs. 20 µM Tyr + DEXA.

The TB assay allowed evaluation of the DEXA effect on our cell model by using only the dosage of 10 µM DEXA, which is the concentration indicated by the literature for obtaining an in vitro atrophic phenotype [20]. After DEXA treatment, myotubes lost their plasma membrane integrity and, if compared to the control condition, showed a significant reduction in cell viability from 90% to 53% (Figure 1A). Later, various Tyr dosages (5 µM, 10 µM, 20 µM, and 40 µM) administrated for 24 h before DEXA exposure were tested in differentiated cells. As shown in Figure 1A, the best Tyr dosage able to counteract in a highly significant manner the DEXA-induced decrease in cell viability was 20 µM. Finally, the TB exclusion assay was also performed for evaluation of the myotube response to 20 µM Tyr, and demonstrated that the compound did not alter cell viability. In fact, myotubes treated with 20 µM Tyr showed a viability percentage comparable to that of control cells (Figure 1B).

Tyr20 alone does not induce a reduction in cell viability, maintaining a percentage similar to the control condition (B). Figure 1 shows ESEM observations (C–E) of control cells (C); myotubes treated with DEXA (D); and myotubes pre-treated with 20 µM Tyr and then exposed to DEXA (E). The mean values of myotube transversal diameter were calculated in all experimental conditions (F). Statistical analyses show a high significance (**) with the *p*-value < 0.005 for ctrl vs. DEXA and DEXA vs. Tyr20 + DEXA. Scale bars: 200 µm for C–E.

### 3.2. ESEM and TEM

**Figure 2:**
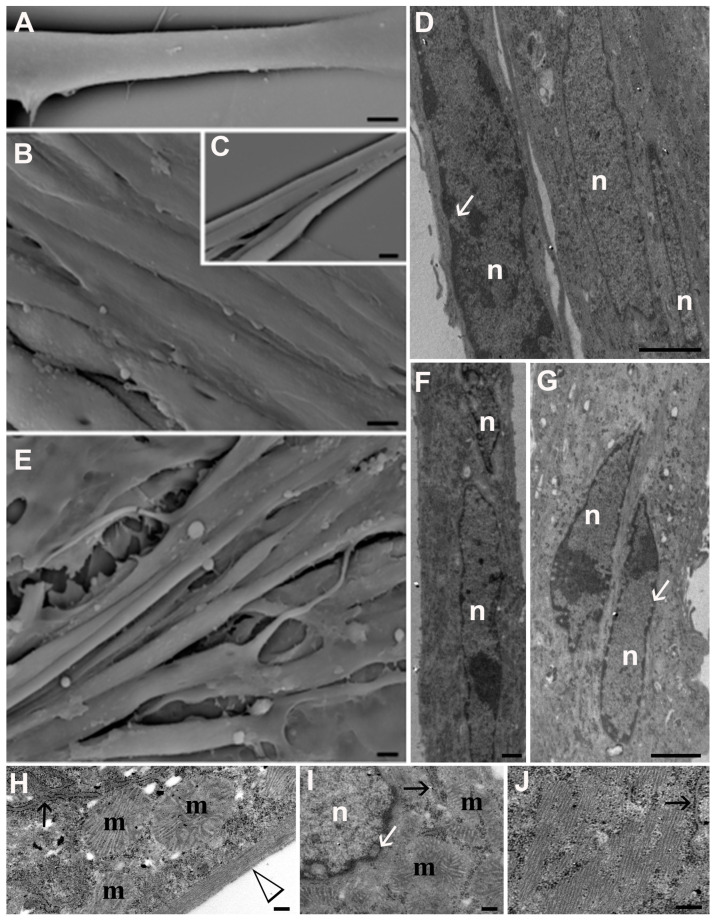
ESEM (**A**–**C**,**E**) and TEM (**D**,**F**,**G**–**J**) micrographs of control cells (**A**–**D**,**H**) and myotubes exposed to 20 µM Tyr (**E**,**F**,**I**,**J**). Bars: 10 µm for (**A**–**C**,**E**); 2 µm for (**D**,**F**,**G**); 200 nm for (**H**–**J**). n: nucleus; m: mitochondria; white arrows indicate nuclear pores; black arrows indicate endoplasmic reticula, and the arrowhead identifies stress fibers.

**Figure 3:**
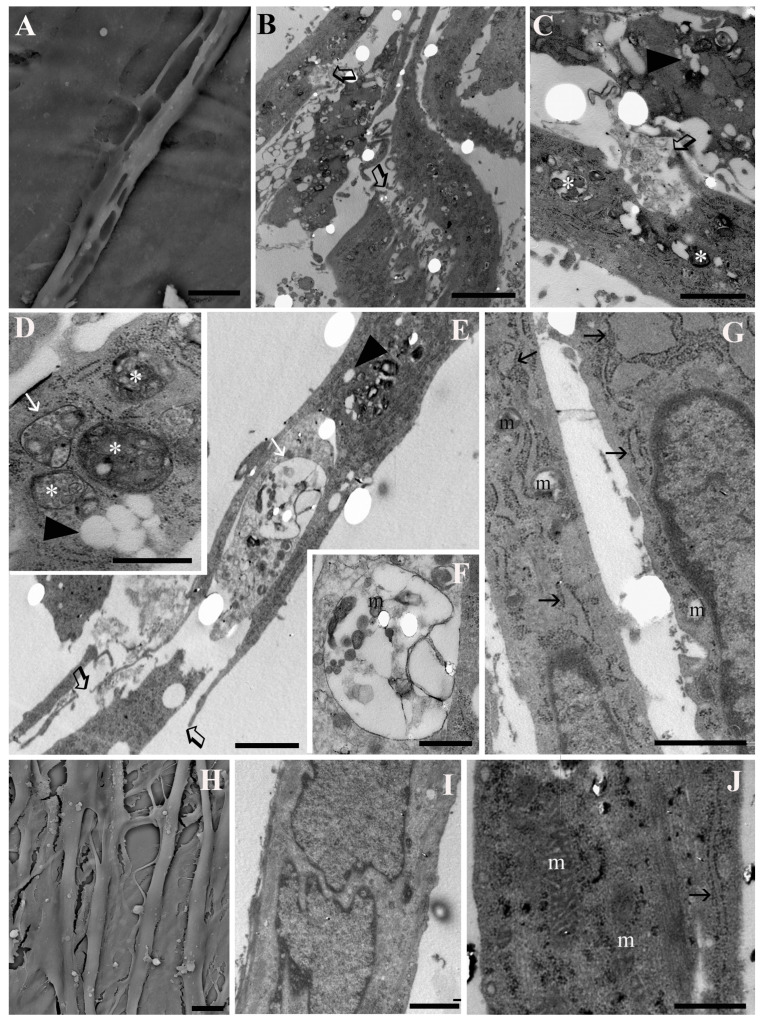
ESEM (**A**,**H**) and TEM (**B**–**G**,**I**,**J**) micrographs of myotubes exposed to DEXA (**A**–**G**) and those pre-treated with 20 µM Tyr before DEXA (**H**–**J**). Open black arrows indicate plasma membrane rupture (**B**,**C**,**E**). Autophagosomes (asterisks) and autophagolysosomes (white arrows) can be observed in (**D**,**E**). Aqueous vacuoles (black arrowheads) appear in the cytoplasm (**C**,**D**). In (**G**,**J**), black arrows refer to endoplasmic reticula. m: mitochondria. Bars: 10 µm for (**A**,**B**); 5 µm for (**C**,**I**,**J**); 2 µm for (**E**,**G**); 20 µm for (**H**); 500 nm for (**D**,**F**).

ESEM observations revealed significant changes in myofiber thickness and length after treatments (Figure 1C–E). At the same magnification, morphological variations in myotube size can be clearly appreciated. After DEXA exposure (Figure 1D), differentiated cells appeared thinner and shorter when compared to those of the control condition (Figure 1C) and to those pre-treated with Tyr before DEXA administration (Figure 1E). The transversal diameter of cells in healthy myotubes was about 18 µm; this drastically and significantly decreased after DEXA treatment to 10 µm, and it was about 15µm when cells were pre-treated with Tyr before DEXA (Figure 1F). Therefore, Tyr appeared able to preserve muscle mass integrity reduced by DEXA, and this is a statistically significant result (Figure 1F). Morphological approaches, thanks to high magnification images, allowed the description of myotube behavior in all experimental conditions and an understanding of the subcellular structures involved in muscle mass loss after DEXA treatment and those that remain functional after Tyr administration before drug exposure (Figure 2 and Figure 3).

From ESEM, control cells showed a smooth surface and intact plasmatic membrane (Figure 2A–C). They were aligned with each other (Figure 2B) and tended to merge to form larger myofibers (Figure 2C). A similar behavior was observed when treating differentiated cells with Tyr alone (Figure 2E). From TEM, control myotubes (Figure 2D,H) and those treated with Tyr (Figure 2F,G,I,J) showed multilobed nuclei (n) localized at the center of the cell (Figure 2D,F,G). The nuclear membrane as well as nuclear pore distribution (white arrows) appeared preserved. Numerous preserved mitochondria (m) surrounded the nuclei and were found scattered in the cytoplasm (Figure 2H,I); stress fibers (arrowhead) appeared organized at the cell periphery (Figure 2H) and presented a parallel distribution at high magnification (Figure 2H,J). Preserved endoplasmic reticula (black arrows) can be identified (Figure 2H–J).

Evident cell shrinkage, diffuse cytoplasm vacuolization (black arrowheads), and general cell damage appeared after DEXA exposure (Figure 3A–G). Both from ESEM and TEM, large vacuoles could be appreciated inside myotube cytoplasm and were often released in the extracellular space, resulting in cell necrosis (Figure 3C–F). In Figure 3B,C,F, open black arrows indicate plasmatic membrane rupture, demonstrating the presence of necrotic features. In the cytoplasm, aqueous vacuoles (black arrowheads) coexisted with degradative vacuoles. The latter contained remnants of cell membranes, altered mitochondria, or degenerated organelles, and their presence suggests activation of the autophagic pathway. Numerous autophagosomes were recognized thanks to their double membrane (white asterisks), and autophagolysosomes with a single membrane (white arrows) could be observed (Figure 3D–F). In F, a high magnification of a degradative vacuole that contains altered organelles and membranes is shown. Mitochondria (m) appeared empty with disorganized cristae, and were often swallowed inside vacuoles (Figure 3E–G). Furthermore, DEXA treatment induced endoplasmic reticulum (ER) stress; indeed, endoplasmic reticula appeared highly dilated (Figure 3G, black arrows), contributing to myotube death. Tyr administration before DEXA exposure (Figure 3H–J) showed a beneficial effect on muscle mass preservation. While some myotubes appeared smaller, the others were similar to the control condition in terms of size, both for the transverse diameter and for length (Figure 3H). Moreover, most of the myotubes preserved their plasma and nuclear membrane integrity (Figure 3I), suggesting that Tyr can prevent DEXA-induced cell death. In a high number of myotubes, ER cisternae (Figure 3J; black arrow) showed a dimension similar to that of control cells; mitochondria (m) preserved their morphology, and a correct cristae disposition could be appreciated (Figure 3J).

### 3.3. CLSM

**Figure 4:**
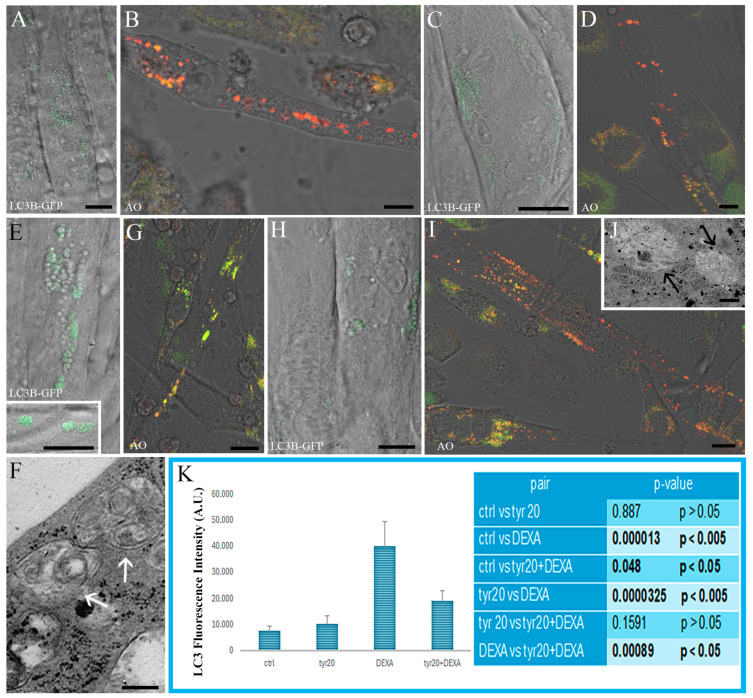
CLSM ((**A**–**E**), inset (**E**,**G**,**H**,**I**)) and TEM (**F**,**J**) micrographs of control cells (**A**,**B**) and myotubes exposed to Tyr alone (**C**,**D**), to DEXA ((**E**), inset (**E**–**G**)), and pre-treated with Tyr before DEXA administration (**H**–**J**). LC3B-GFP ((**A**,**C**,**E**), inset (**E**,**H**)) and AO staining (**B**,**D**,**G**,**I**) can be observed. LC3B green puncta are associated with autophagosomes; vacuoles are characterized by a double membrane (white arrows, in (**F**)) while autophagolysosomes (black arrows in (**J**)) appear in myotubes pre-treated with Tyr before DEXA exposure. AO is accumulated by acidic vesicles that, if functional, yield prominent orange or red signal staining. When the lysosomal proton gradient is lost, leakage of AO into the cytosol can be detected as a concomitant increase in green cytosolic staining. Graph (**K**) shows the fluorescence intensity quantification of LC3 green puncta (with relative Tukey HSD values and significant differences among the various experimental groups). Bars: 10 µm for ((**A**–**E**), inset (**E**,**G**,**H**,**I**)); 200 nm for (**F,J**).

**Figure 5:**
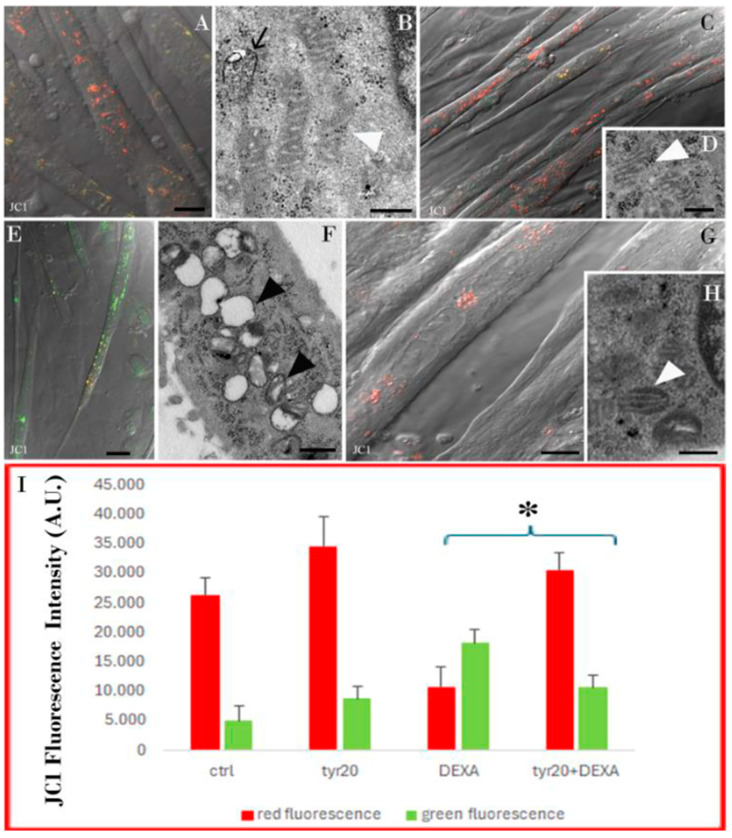
CLSM (**A**,**C**,**E**,**G**) and TEM (**B**,**D**,**F**,**H**) micrographs of control cells (**A**,**B**), and myotubes exposed to Tyr alone (**C**,**D**), to DEXA (**E**,**F**), or pre-treated with Tyr before DEXA administration (**G**,**H**). JC1 staining emits red fluorescence when the mitochondrial membrane potential is preserved (**A**,**C**,**G**), whereas it emits green fluorescence in unhealthy myotubes (**E**). Preserved ((**B**,**D**,**H**); white arrowheads) or altered ((**F**), black arrowheads) mitochondria can be observed at ultrastructural levels. In (**B**), a degrative vacuole at a single membrane appears (black arrow). Graph (**I**) shows the fluorescence intensity quantification of JC1 staining calculated with ImageJ Software. JC1 immunofluorescence quantification appears statistically significant with the *p*-value < 0.05 (*) when comparing DEXA alone vs. Tyr20 + DEXA treatments. Bars: 10 µm for (**A**,**C**,**E**,**G**); 200 nm for (**B**,**D**,**F**,**H**).

Some functional analyses using CLSM were performed to evaluate autophagy and lysosomal compartment activity (Figure 4) as well as mitochondria functionality (Figure 5). Control cells and those exposed only to Tyr showed a low number of autophagosomes, as evidenced by a low density of LC3-stained puncta (Figure 4A,C). Furthermore, functional lysosomes, detected thanks to AO red puncta (Figure 4B,D), and mitochondria with membrane potential preservation (Figure 5A,C) could be observed in both experimental conditions. This scenario was confirmed after TEM observations where myotubes mainly showed vacuoles with a single membrane (black arrow) and mitochondria with a preserved morphology (Figure 5B,D). The situation changed after DEXA treatment. A high number of autophagosomes (Figure 4E, inset E) identified using TEM, thanks to their double membranes (Figure 4F), appeared inside myotubes together with dysfunctional lysosomes (Figure 4G). If, in control cells, most of the AO was identified inside the lysosomes as punctuated red patterns (Figure 4B), after DEXA exposition, an increase in green fluorescence appeared (Figure 4G), suggesting a loss of lysosomal membrane integrity, which, as known, leads to cell death induction. In addition, after DEXA treatment, mitochondria (Figure 5E) lost their membrane potential and became, as revealed also by means of TEM, empty with an irregular cristae disposition (Figure 5F, arrowheads). Tyr administration before DEXA was able to rescue correct autophagic flux in most cells (Figure 4H–J), characterized by the presence of scarce autophagosomes (Figure 4H) and functional lysosomes (I); in fact, mainly single-membrane degradative vacuoles were detected using TEM (Figure 4J). Most mitochondria appeared functional (Figure 5G) and reacquired their preserved morphology (Figure 5H). Fluorescence intensity quantification confirmed this behavior (Figure 4K and Figure 5I), and analyzing the fluorescence intensities of LC3 (Figure K) and JC1 (Figure 5I) staining showed that Tyr pre-treatment before DEXA exposure restored autophagic flux and mitochondria membrane potential. These findings are statistically significant.

## 4. Discussion

Skeletal muscle represents almost 40% of total body mass and plays a crucial role in thermoregulation, systemic metabolism, exercise, and visceral protection [27]. Consequently, maintenance of skeletal muscle homeostasis is vital for preventing metabolic disorders and promoting healthy aging [28,29].

Skeletal muscle mass loss represents a crucial health problem worldwide, especially in older people. It is known that oxidative stress and inflammation are two crucial factors involved in muscle atrophy development [3]. Physiological and pathological conditions, such as aging, chronic diseases, cachexia, disuse, and denervation, are characterized by high levels of oxidative stress, which lead to proteolytic pathway activation and to mitochondrial dysfunctions [11]. These pathological conditions are also associated with elevated circulating glucocorticoid levels [27].

Glucocorticoids are steroid hormones with therapeutic anti-inflammatory and immunosuppressive activities; however, they show adverse effects, which also include skeletal muscle wasting [27]. DEXA is a long-acting synthetic glucocorticoid able to suppress protein synthesis and to induce muscle atrophy by involving the oxidative-stress-mediated pathway [30].

Taken together, these considerations suggest that preventing and attenuating skeletal muscle atrophy appears to be an important clinical goal. To date, nutritional approaches for treating muscle wasting include protein intake, supplementation with antioxidants, essential amino acids and omega-3 fatty acids, and other food interventions [30].

Here, we demonstrated Tyr’s beneficial effect in delaying muscle mass loss and in preventing myotube wasting induced by a glucocorticoid drug usually used in vitro to simulate muscle atrophy. Tyr is one of the simplest phenols found in virgin olive oil and investigating its cellular effects appears to be an interesting topic. Tyr shows unique biological properties, acting, “in primis”, as an effective and powerful antioxidant [15]. Recent studies described its potential antigenotoxic activity, and its effectiveness in the prevention of apoptosis in keratinocytes [15], in the preservation of endothelial dysfunction, in ameliorating hyperglycemia in rats, and in reducing oxidative stress in muscle cells [31,32]. Its health benefits associated with the regular consumption of virgin olive oil have been widely demonstrated in different models both in vivo and in vitro. In this regard, Tyr acts as cardioprotective, antiviral, anti-inflammatory, neuroprotective, and anticancer compound [13,33,34,35,36,37,38].

Numerous in vitro and in vivo studies have reported polyphenols as strongly effective bioactive compounds able to enhance muscle health and to counteract muscle wasting, including the one induced by glucocorticoids. In this context, different polyphenols, such as quercetin, glabidrin, resveratrol, and oligonol, can effectively attenuate DEXA-induced muscle atrophy [39,40,41,42,43,44,45,46]. The novelty of this manuscript allows us to consider Tyr as a beneficial compound able to contribute to skeletal muscle homeostasis preservation against DEXA-induced myotube mass loss. In fact, no data on Tyr’s effect on skeletal muscle wasting is available. Here, we report the effect of this phenolic molecule on C2C12 myotubes exposed to DEXA, which is an atrophic in vitro drug. Morpho-functional analyses demonstrated that Tyr recovers, in a statistically significant manner, myotube diameter and maintains muscle mass by improving lysosome and mitochondria function, and, consequently, by balancing cell death and autophagic processes. As known, autophagy is a crucial cellular process involved in removing damaged organelles and myofibers as well as aggregated or misfolded proteins inside double membrane structures. When this content is degraded, autophagy promotes muscle regeneration and remodeling after injury [27,47]. However, its aberrant activation or impairment leads to muscle alterations, which contribute to muscle atrophy. Here, we demonstrated that after DEXA treatment myotubes degenerated with the involvement of endoplasmic reticulum stress, mitochondria alteration, and autophagosome accumulation; these are all conditions that finally lead to necrotic cell death. DEXA-treated myotubes after TEM observations show cytoplasmic accumulation of autophagosomes and large autophagolysosomes that do not re-emerge in their process of degradation and induce necrotic cell death. In fact, a high percentage of myotubes appear dead after both the TB assay and ultrastructural analyses.

In this regard, CLSM observations showed a conspicuous number of autophagosomes together with dysfunctional lysosomes, demonstrating an impairment of the autophagic degradative pathways after DEXA exposure. As known, destabilization of the lysosome compartment often proceeds necrotic cell death. Furthermore, the presence of degenerated mitochondria contributes to myotube damage. Excessive ROS production promotes mitochondrial degradation and mitochondrial membrane potential impairment [48,49], which is a condition that occurred in our experiments treating myotubes with DEXA. The presence of dysfunctional mitochondria after DEXA treatment was followed by toxic protein accumulation inside C2C12 differentiated cells due to an impairment of autophagy processes, which contributed to oxidative stress, leading to necrotic cell death. Thus, oxidative stress is strictly connected with mitochondrial damage/autophagy [50]. In this scenario, Tyr administration before DEXA maintained a correct balance between cell death and autophagy, which is an essential condition that contributes to the preservation of skeletal muscle mass and homeostasis [51]. After Tyr exposure, cytoplasmic organelles reacquired a preserved morphology and rescued their functionality, which are essential for maintaining the quality of skeletal muscle. Most myotubes pre-treated with Tyr before DEXA have an intact plasmatic membrane, mitochondria with a regular cristae disposition, few and small degradative vacuoles, functional lysosomes, and preserved mitochondrial membrane potential. This result indicates a crucial role of Tyr in the maintenance of autophagy-lysosomal machinery and mitochondrial activity, which are indispensable conditions for preventing muscle atrophy. In fact, as known, mitochondrial functionality and the autophagy pathway are two critical processes involved in cell protection against oxidative stress and against the decline that occurs, for instance, with aging [51].

Therefore, in a C2C12 differentiated cell line, which is a model widely used to study in vitro muscle development and atrophy, Tyr can be considered an effective intervention against glucocorticoid-induced muscle wasting by promoting mitochondria homeostasis and maintaining a correct autophagic flux.

## 5. Conclusions

This is the first morpho-functional study that evaluated the effect of Tyr against muscle atrophy induced in C2C12 myotubes in vitro.

Taken together, these data demonstrate a potential role of Tyr as an innovative and promising therapeutic strategy against skeletal muscle degenerative conditions, and highlight the need for additional studies aimed to further investigate the molecular targets involved when Tyr orchestrates muscle homeostasis preservation. Therefore, increasing Tyr knowledge appears relevant to the scientific community and opens up new scenarios for novel investigations on the role of Tyr in preclinical and clinical studies as a dietary supplement to be used in synergy with other functional foods or with exercise training.

## Data Availability

Data is contained within the article.
